# Robotic-assisted transbronchial biopsy versus computed tomography–guided transthoracic needle biopsy for peripheral pulmonary lesions: a systematic review and meta-analysis of direct comparative studies

**DOI:** 10.1007/s11701-026-03867-8

**Published:** 2026-09-04

**Authors:** Atef A. Hassan, Mohamed Mostafa El Sheikh, Amr A. Hassan, Abdelrahman Mady, Mohab Hesham, Yousef Mohamed Hamroush, Shimaa Mostafa Elnahas Wahdan, Mustafa M. Kaff, Mohamed Ashraf Abd El Moniem, Mona Ibrahim Ahmed

**Affiliations:** 1 Faculty of Medicine, Al-Azhar University, Cairo, Egypt; 2 Chest Department, Faculty of Medicine, Kafr El Sheikh University, Kafr El Sheikh, Egypt; 3 Department of Diagnostic and Interventional Radiology, Faculty of Medicine, Al-Azhar University, Cairo, Egypt; 4 Department of Diagnostic and Interventional Radiology, Faculty of Medicine, Horus University, New Damietta, Egypt; 5 Chest Department, Faculty of Medicine, Benha University, Benha, Egypt; 6 Department of Diagnostic and Interventional Radiology, Faculty of Medicine, Al-Azhar University, New Damietta, Egypt; 7 Chest Department, Faculty of Medicine, Fayoum University, Fayoum, Egypt

**Keywords:** Robotic-assisted bronchoscopy, Transthoracic needle biopsy, Peripheral pulmonary lesion, Lung cancer, Diagnostic yield, Pneumothorax, Meta-analysis

## Abstract

Robotic-assisted bronchoscopy (RAB) and computed tomography–guided transthoracic biopsy (CTTB) are competing strategies for sampling peripheral pulmonary lesions (PPLs). Whether they differ in yield or safety is uncertain. To our knowledge, this is the first systematic review restricted to direct comparisons. We searched MEDLINE, Europe PMC, Scopus, Web of Science and ClinicalTrials.gov from inception to 7 July 2026 for studies directly comparing RAB with CTTB in adults with PPLs. The primary outcome was strict 2024 American Thoracic Society/American College of Chest Physicians diagnostic yield. Risk of bias was assessed with ROBINS-I and certainty with GRADE. A cohort-genealogy step identified, per outcome, the largest set of cohorts sharing no patients; only that set was pooled, with Hartung–Knapp and Mantel–Haenszel sensitivity analyses. Five retrospective studies from one US health system were eligible. Four share patients; at most three cohorts are mutually independent. Across those three, diagnostic yield was comparable (risk ratio [RR] 0.99, 95% confidence interval [CI] 0.93–1.06; I²=24%; Hartung–Knapp 0.87–1.13), with an identical relative effect under strict and intermediate definitions although absolute yields fell from 88% to 74–84% under strict criteria. Pneumothorax requiring a chest tube and/or admission was about three-quarters less frequent with RAB across all three cohorts (RR 0.25, 95% CI 0.14–0.46; I²=0%; Hartung–Knapp 0.07–0.96). Strict yield (RR 0.99) and any pneumothorax (RR 0.06) were reported by two cohorts each and neither survives the few-studies correction. RAB took about 50 min longer than CTTB where same-session staging endobronchial ultrasound was counted in the robotic time, but only about 8 min longer than CTTB where it was not. Only one cohort reported yield by lesion size category and none reported yield by bronchus sign or lung zone, so lesion-level subgroups could not be pooled. Certainty was low for pleural complications and very low elsewhere. Low-certainty evidence indicates that RAB is associated with fewer pleural complications, with no statistically detectable difference in diagnostic yield; equivalence was not formally established. Because all evidence is retrospective, confined to one health system, and almost never stratified by lesion size or accessibility, these findings are hypothesis-generating and require a multicenter randomized trial.

**Supplementary Information:**

The online version contains supplementary material available at https://doi.org/10.1007/s11701-026-03867-8.

## Introduction

Lung cancer remains the leading cause of cancer-related death worldwide [1]. The expansion of low-dose computed tomography (CT) screening and the incidental detection of pulmonary nodules on cross-sectional imaging have produced a large and growing population of patients who require evaluation of peripheral pulmonary lesions (PPLs) [2, 3]. Because the majority of screen- or incidentally-detected nodules are ultimately benign, accurate and safe tissue sampling is central to avoiding both delayed cancer diagnosis and unnecessary intervention [4–6].

Two minimally invasive strategies dominate nonsurgical sampling of PPLs. CT-guided transthoracic biopsy (CTTB) has historically offered high diagnostic accuracy but carries a well-documented risk of pleural complications, with pneumothorax reported in up to a quarter of procedures and chest-tube placement in a clinically important minority [7, 8]. Bronchoscopic sampling avoids transpleural needle passage but has traditionally suffered from lower and highly variable diagnostic yield [9, 10]. Navigational technologies have since been introduced to close this gap: electromagnetic navigation bronchoscopy improved bronchoscopic reach [11, 12], and shape-sensing robotic-assisted bronchoscopy (RAB) platforms further enhance catheter stability, distal control, and tool-in-lesion accuracy while preserving the option of same-session mediastinal staging [13–15].

Single-arm cohorts and modality-specific meta-analyses report favorable RAB diagnostic performance and low complication rates [16–19], but they cannot establish how RAB compares with the established percutaneous standard. Direct head-to-head comparisons are scarce, and their interpretation has been hampered by inconsistent definitions of “diagnostic yield,” with many studies counting nonspecific or follow-up-informed results as diagnostic and thereby inflating apparent performance [20, 21]. To address this, a 2024 ATS/ACCP research statement proposed a strict, index-procedure definition in which only a specific malignant or specific benign diagnosis is considered diagnostic [20]. A prior network meta-analysis compared multiple biopsy modalities but derived the RAB-versus-CTTB contrast almost entirely from indirect evidence [22], and recent randomized trials in the navigational-bronchoscopy space—VERITAS and RELIANT—did not compare a robotic platform directly with CTTB [23, 24].

No systematic review has synthesized direct RAB-versus-CTTB evidence under a strict diagnostic-yield definition or addressed the risk of patient double-counting from overlapping reports. Therefore, we conducted a systematic review and meta-analysis with a cohort-genealogy step to estimate the effectiveness, safety, and outcomes of RAB versus CTTB for PPLs.

## Methods

### Protocol, registration, and reporting

The review followed a written protocol completed before the searches were run (version 1.0, 6 July 2026), prepared in accordance with the PRISMA-P 2015 statement [25] and provided in full as a supplementary file. It is reported in accordance with the PRISMA 2020 statement [26] and, because non-randomized studies were pooled, with reference to the MOOSE guidance for meta-analysis of observational studies [27]; the completed PRISMA 2020 checklist is supplied. The protocol has been submitted to PROSPERO and the registration number will be provided to the editorial office as soon as it is issued; the review was therefore not prospectively registered at the time the searches were run, which we state explicitly rather than describe the review as registered. Deviations from the protocol are listed at the end of the Methods and no other amendments were made.

### Information sources, search, and study selection

We searched MEDLINE (via PubMed), Europe PMC, Scopus, and the Web of Science Core Collection, together with the ClinicalTrials.gov trials register, from database inception to 7 July 2026, without language or publication status restrictions. The search combined three concept blocks — robotic-assisted bronchoscopy, CT-guided transthoracic/percutaneous biopsy, and pulmonary lesion/nodule — using controlled vocabulary and free-text terms adapted to each platform. Reference lists of included studies and relevant reviews were hand-searched and forward-citation searches were performed; these methods identified no additional eligible records. Titles, abstracts and full texts were screened by one reviewer and independently checked by a second, with disagreements resolved by consensus and a third reviewer available for adjudication. The complete, line-by-line search strategy for every source, with the exact syntax and the number of records retrieved, is provided in **Supplementary Table 1**.

### Eligibility criteria

We included randomized trials and comparative prospective, retrospective, or propensity-matched cohort studies that directly compared RAB (any commercial platform) with CTTB (including CT-fluoroscopy–guided percutaneous transthoracic biopsy) in adults (≥ 18 years) undergoing sampling of a PPL, and that reported at least one extractable comparative outcome. We excluded single-arm reports, studies without a CTTB comparator, studies of central or mediastinal lesions only, and conference abstracts (eligible only for a prespecified sensitivity analysis that could not be performed; see Deviations from the protocol). Where a study selected patients solely on a final cancer diagnosis, it was eligible only for secondary, cancer-specific analyses.

### Data extraction and cohort-overlap management

One reviewer extracted study, participant, lesion, procedural and outcome data using a piloted form, and a second reviewer independently verified every extracted value against the source article, with disagreements resolved by discussion. Study authors were not contacted for additional or unpublished data. Because multiple reports from a single health system share centres and recruitment windows, we constructed a prespecified cohort-genealogy table (institution, campus, recruitment period, sample size, diagnostic-yield definition, and cohort role). When cohorts overlapped or overlap could not be confidently excluded, only one report per overlapping cohort contributed to any given pooled estimate, selected by strictness of the yield definition, completeness of event data, and lower risk of bias.

### Outcomes and definitions

The primary outcome was strict diagnostic yield, defined per the 2024 ATS/ACCP statement as the proportion of attempted or performed index procedures yielding a specific malignant or specific benign diagnosis; atypia, nonspecific inflammation, and insufficient tissue were classified as nondiagnostic, and follow-up results were not used to reclassify an initially nondiagnostic result [20]. Secondary outcomes included any pneumothorax, chest-tube placement, hospital admission, pulmonary hemorrhage, molecular/genomic adequacy, procedure duration, time to treatment, same-session mediastinal staging, stage at diagnosis, and procedure-related mortality.

### Risk of bias and certainty of evidence

Risk of bias was assessed with ROBINS-I for non-randomized studies of interventions [28], by one reviewer and independently verified by a second, with disagreements resolved by consensus. Prespecified critical confounders were lesion size and density, pleural distance, bronchus sign, emphysema, and pre-test malignancy probability. Certainty of evidence for each outcome was rated using GRADE [29].

### Statistical analysis

Risk ratios (RRs) were the effect measure for dichotomous outcomes and mean differences (MDs) for continuous outcomes; study-level effects and variances were computed on the log scale for ratio measures. Where a continuous outcome was reported as a median with interquartile range, the mean was derived by the method of Luo and colleagues and the standard deviation by the method of Wan and colleagues [30, 31]. For every outcome we first identified the largest set of mutually independent cohorts permitted by the cohort-genealogy graph and pooled only that set; one outcome could therefore draw on a different representative of an overlapping lineage than another, but no pooled estimate ever combined two cohorts that share patients. The protocol prespecified a random-effects primary synthesis. Because at most three independent cohorts contributed to any outcome, that analysis was supplemented by a post hoc hierarchy of models: inverse-variance fixed-effect, DerSimonian–Laird random-effects [32], random-effects with the Hartung–Knapp–Sidik–Jonkman (HKSJ) variance correction [33] applied with the Rover ad-hoc variance floor, so that the corrected interval can never be narrower than the DerSimonian–Laird interval [34], and Mantel–Haenszel pooling for the low-event safety outcomes [35]. Heterogeneity was quantified with Cochran’s Q, I² and τ². No study contained a zero-event cell, so no continuity correction was applied. Two subgroup analyses were specified once the eligible cohorts were known and are reported as post hoc: for diagnostic yield, strict versus intermediate yield definition; for procedure duration, whether same-session staging endobronchial ultrasound was counted in the robotic time. Because fewer than ten cohorts contributed to any outcome, funnel-plot and small-study-effect tests and prediction intervals were not computed, consistent with Cochrane guidance [36]. The HKSJ and Mantel–Haenszel models and both subgroup analyses were not named in the protocol and are reported as post hoc methodological safeguards. Analyses were performed in Python 3.12 (NumPy, SciPy).

### Deviations from the protocol

Three deviations occurred and are reported in full. First, the protocol prespecified searches of MEDLINE, Embase, Web of Science Core Collection, Scopus and Cochrane CENTRAL. Embase and CENTRAL were not accessible to the review team; Europe PMC, which indexes MEDLINE, PubMed Central and preprints, and the ClinicalTrials.gov register were searched in their place. This is a genuine limitation and we do not claim an exhaustive search. Its likely impact is small but not nil: CENTRAL indexes controlled trials, and no randomized comparison of the two modalities exists, so that database could not have contributed an eligible study; Embase indexes conference proceedings more completely than MEDLINE, so the most plausible loss is an unpublished abstract rather than a full report. Backward- and forward-citation searching of all five included studies and of the three most recent relevant reviews identified no eligible study outside those five. Second, three prespecified sensitivity analyses — inclusion of conference abstracts, exclusion of studies at serious or critical risk of bias, and a cluster-aware re-analysis of the lesion-level cohort — could not be performed, because after resolution of cohort overlap at most three independent cohorts remained and each analysis would have left fewer than two poolable cohorts. Third, the Hartung–Knapp and Mantel–Haenszel models, the Röver variance floor and the two subgroup analyses were not named in the protocol and are reported as post hoc methodological safeguards. Of the outcomes listed for GRADE assessment in the protocol, repeat biopsy was not reported in a comparable form by any independent cohort and is not included in the Summary of Findings.

## Results

### Study selection and characteristics

**Fig. 1:**
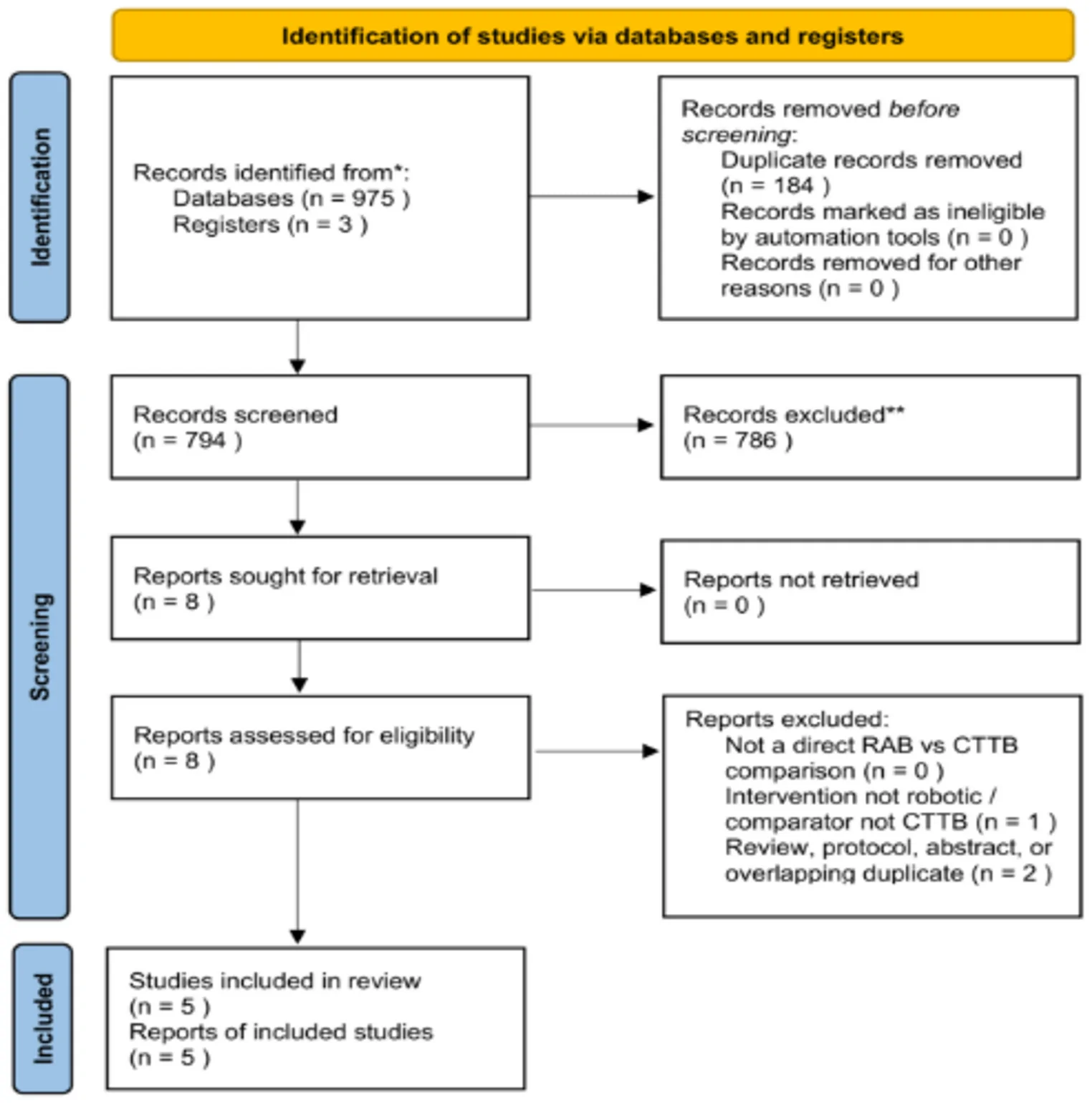
PRISMA 2020 flow diagram of study identification, screening, and inclusion. Counts reflect the database and register searches executed on 7 July 2026: MEDLINE (via PubMed), Europe PMC, Scopus, Web of Science, and the ClinicalTrials.gov register; full strategies and per-database record counts are given in Supplementary Table 1. *Records identified from databases and registers only: reference-list and forward-citation searching yielded no additional records, so no studies were identified by other methods and the PRISMA 2020 “other methods” column is not shown. **Records excluded by a human reviewer on title and abstract; no automation tools were used. CTTB, CT-guided transthoracic biopsy; RAB, robotic-assisted bronchoscopy

Database and register searches, run on 7 July 2026, identified 975 records through database searching (MEDLINE via PubMed, *n* = 95; Europe PMC, *n* = 69; Scopus, *n* = 736; Web of Science, *n* = 75) and 3 records through the ClinicalTrials.gov register. After 184 duplicate records were removed, 794 records were screened by title and abstract; 786 were excluded as clearly ineligible (predominantly narrative reviews and meta-analyses, single-arm robotic-bronchoscopy series, ablation and nodule-localization studies, and off-topic robotic-intervention reports). Eight reports were assessed for eligibility in full text; three were excluded — two conference abstracts and one comparing cone-beam CT–guided bronchoscopy, rather than a robotic platform, with CTTB. The first abstract compared RAB with CTTB but was identified as a duplicate report of the included DYSTRICT cohort (same campus, recruitment window, lesion count and authorship) and was therefore excluded as a duplicate rather than on abstract status alone; the second compared shape-sensing RAB with transthoracic needle aspiration. **Five** studies met all eligibility criteria and were included (Fig. 1).

The search identified five eligible direct-comparison studies, all retrospective and all originating from a single US health system (Mayo Clinic) [37–41]. No independent, non–Mayo Clinic study directly comparing RAB with CTTB was identified. Summed across their analysed cohorts the five reports describe 1,783 participants and 1,799 sampled lesions (Table 1); because four reports draw on overlapping institutional cohorts, the number of unique patients is smaller and cannot be determined from the published data. All robotic procedures used the shape-sensing Ion platform, frequently combined with radial endobronchial ultrasound, cone-beam CT or three-dimensional fluoroscopy, rapid on-site evaluation, and cryobiopsy; CTTB was performed percutaneously under CT or CT-fluoroscopic guidance. Study characteristics and baseline participant/lesion data are summarized in Tables 1 and 2.

**Table 1:** Characteristics of the included studies

Characteristic	Lee-Mateus 2023	Fernandez-Bussy 2024 (subsolid)	Fernandez-Bussy 2025 (pathway)	DYSTRICT 2026	McNierney 2026
Reference (PMID/DOI)	PMID 36,104,312/ https://doi.org/10.1111/resp.14368	PMID 38,471,496/ https://doi.org/10.1159/000538132	PMID 40,602,203/ https://doi.org/10.1016/j.lungcan.2025.108646	PMID 42,094,232/ https://doi.org/10.1183/23120541.00895-2025 (PMC13139926)	PMID 42,275,984/ https://doi.org/10.1016/j.lungcan.2026.109487
Country and centre(s)	USA; Mayo Clinic (2 sites) — Florida (Jacksonville) + Rochester	USA; Mayo Clinic (2 sites) — Florida (Jacksonville) + Rochester	USA; Mayo Clinic (single institution) — Florida (Jacksonville)	USA; Mayo Clinic (single center) — Jacksonville, FL	USA; Mayo Clinic (single center) — Phoenix, AZ
Recruitment period	Jan 2019 – Mar 2021	Feb 2020 – Apr 2023	CTTB 2017–2020; ssRAB 2020–2023	Nov 2022 – Dec 2024	21 Mar 2023–31 Aug 2024
Study design	Multicenter retrospective cohort (no formal matching)	Retrospective cohort, subsolid nodules only; propensity-score-weighted regression	Retrospective cohort, 1:1 propensity-matched; cancer/staging pathway	Retrospective cohort, 1:1 propensity-matched (lesion-level)	Retrospective observational; crude full-cohort comparison + genetic 1:1 PSM (with replacement) for adjusted analysis
Sample size, n (RAB/CTTB)	113/112	27/38	148 (matched; 556 pre-match)/148 (matched; 407 pre-match)	287 nodules (272 pts; 845 pre-match)/287 nodules (287 pts)	489/149
Unit of analysis	Patient/nodule (225)	Patient (65)/nodule (66)	Patient	Nodule/lesion	Patient (peripheral lesion; multi-lesion encounters excluded)
RAB platform	Ion Endoluminal System (Intuitive Surgical)	Ion Endoluminal System (Intuitive Surgical)	Ion Endoluminal System (Intuitive Surgical)	Ion Endoluminal System (Intuitive Surgical)	Ion Endoluminal System (Intuitive Surgical)
CTTB technique	CT-guided (SOMATOM, Siemens); coaxial core biopsy; needle 18–21G	CT-guided (SOMATOM, Siemens); local anesthesia	CT-guided transthoracic biopsy	CT-guided, fluoroscopic; local anesthesia; ROSE available	CT-guided (interventional radiology); moderate sedation; 19G introducer + 20G Temno core
Matching/adjustment	None (crude subgroup comparison; PTX model adjusted)	Propensity-score weighting (no 1:1 match)	1:1 PSM (MatchIt); 148 per group	1:1 propensity-score matching (age, sex, size, type, location)	Genetic 1:1 PSM with replacement (489 matched pairs) for adjusted estimates
Diagnostic-yield definition	Intermediate (accuracy-based, 12-mo follow-up; ‘suspicious’ counted)	Intermediate (diagnostic accuracy at 6–12 mo; ‘suspicious’ counted)	Malignancy diagnostic yield (cancer-only, follow-up-confirmed)	STRICT (ATS/ACCP; day-of-procedure histopathology only)	STRICT (ATS/ACCP research statement)
Funding; conflicts of interest	No external funding stated; COI: None declared	No funding required (stated); COI: None declared	No external financial support (stated); COI: None declared	No funding statement; COI: None declared	No funding statement; COI: None declared
Role in this review	INDEPENDENT COHORT 1 of 3. Pools for study-defined yield, pneumothorax requiring intervention and procedure duration. Recruitment window is disjoint from DYSTRICT, so the two share no patients	NOT POOLED. Overlaps Lee-Mateus, Fernandez-Bussy 2025 and DYSTRICT; subsolid nodules only; reported yields are internally irreconcilable. Narrative only	NOT POOLED for yield or safety (overlaps DYSTRICT; cancer-selected cohort). Contributes to the time-to-treatment pool, where it is independent of McNierney	INDEPENDENT COHORT 2 of 3. Pools for every yield, safety and duration outcome; the Jacksonville representative of the Florida lineage	INDEPENDENT COHORT 3 of 3. Pools for every outcome; the only non-Florida cohort and independent of all four others

CTTB, CT-guided transthoracic biopsy; COI, conflicts of interest; DY, diagnostic yield; EBUS-TBNA, endobronchial ultrasound transbronchial needle aspiration; NR, not reported; PSM, propensity-score matching; RAB, robotic-assisted bronchoscopy; rEBUS, radial endobronchial ultrasound; ssRAB, shape-sensing RAB. Strict diagnostic yield follows the 2024 ATS/ACCP research statement. Cohorts are labelled independent when they share neither a Mayo Clinic campus nor an overlapping recruitment window; at most three of the five reports are mutually independent, and a given pooled estimate uses only mutually independent cohorts

**Table 2:** Baseline characteristics of participants and target lesions

Variable (RAB/CTTB)	Lee-Mateus 2023	Fernandez-Bussy 2024	Fernandez-Bussy 2025	DYSTRICT 2026	McNierney 2026
No. analysed	113 112	27 38	148 (matched) 148 (matched)	272 (287 nodules) 287	489 149
Age	70.0 (mean ± 9.8) y 67.4 (mean ± 12.4) y	71 (median; IQR 64–78) y 69 (median; IQR 61–78) y	71 (median; IQR 65–77) y 72 (median; IQR 65–78) y	69 (median; IQR 62–76) y 69 (median; IQR 61–76) y	70 (median; IQR 61–77) y 70 (median; IQR 63–76) y
Male, n (%)	57 (50.4%) 57 (50.9%)	11 (40.7%) 12 (31.6%)	72 (48.6%) 76 (51.4%)	162 (56%) 155 (54%)	246 (50.3%) 86 (57.7%)
BMI (kg/m²)	27.9 ± 6.5 27.0 ± 5.2	26 (IQR 22.5–28.4) 25.2 (IQR 22.66–29.4)	26 (median) 26.2 (median)	26.5 (IQR 23.3–30.6) 26.4 (IQR 23.1–30.2)	26.1 (IQR 22.9–29.8) 25.3 (IQR 23.1–29.6)
Smoking status	Former 70 (61.5%)/Never 30 (26.5%)/Current 13 (11.5%) Former 57 (50.9%)/Never 47 (42.0%)/Current 8 (7.1%)	Former 20 (74.1%)/Never 5 (18.5%)/Current 2 (7.4%) Former 27 (71.1%)/Never 10 (26.3%)/Current 1 (2.6%)	Former 116 (78.4%)/Never 18 (12.2%)/Current 14 (9.5%) Former 114 (77.0%)/Never 18 (12.2%)/Current 16 (10.8%)	Former 155 (54%)/Never 115 (40%)/Current 17 (6%) Former 141 (49%)/Never 135 (47%)/Current 11 (4%)	Former 245 (50.1%)/Never 215 (44.0%)/Current 29 (5.9%) Former 71 (47.7%)/Never 65 (43.6%)/Current 13 (8.7%)
Prior cancer, n (%)	54 (47.8%) 77 (68.8%)	12 (44.4%) 23 (60.5%)	Lung 34 (23.0%); other 63 (42.6%) Lung 33 (22.3%); other 56 (37.8%)	168 (59%) 248 (86%)	NR NR
Nodule size	1.8 cm (IQR 1.3–2.7) 1.6 cm (IQR 1.0–2.6)	Subsolid only; overall max 1.5 cm; PSN solid comp. 5 mm Subsolid only (see RAB)	20 mm (IQR 13.17–28) 21.25 mm (IQR 13.55–31)	1.50 cm (IQR 1.03–2.39) 1.60 cm (IQR 1.10–2.80)	19 mm (IQR 13–29) 17 mm (IQR 11–29)
Solid nodule, n (%)	91 (81.3%) 98 (87.5%)	0 (subsolid cohort) 0 (subsolid cohort)	84 (56.8%) 119 (80.4%)	220 (77%) 213 (74%)	NR NR
Distance to pleura/chest wall	1.5 cm (IQR 0.6–3.3) 1.3 cm (IQR 0.1–2.3)	2.15 cm to pleura (overall; IQR 1.02–3.70) See overall	13.8 mm to chest wall (IQR 4.4–27.4) 10.02 mm to chest wall (IQR 0–21.9)	1.26 cm to chest wall (IQR 0–2.50) 0.61 cm to chest wall (IQR 0–1.50)	NR (matched on pleura proximity) NR

Top line = RAB arm; bottom line = CTTB arm. BMI, body mass index; IQR, interquartile range; NR, not reported. For DYSTRICT the values are the 1:1 propensity-matched analytic cohort; for McNierney they are the full unmatched cohorts on which the primary comparison was reported. Every cell was verified against the published table of the source article; cells marked NR are not reported in the source. Lee-Mateus reports age and BMI as mean (SD) and the remaining studies as median (IQR)

### Cohort overlap

The cohort-genealogy assessment treated two reports as overlapping when they shared a Mayo Clinic campus and their recruitment windows intersected. On that rule the four Florida/Rochester reports form a single connected cluster: Lee-Mateus overlaps Fernandez-Bussy 2024 and Fernandez-Bussy 2025, and Fernandez-Bussy 2024 and 2025 each overlap DYSTRICT [37–40]. The Phoenix cohort is independent of all four [41]. Two members of the cluster do not overlap each other directly, however: Lee-Mateus recruited from January 2019 to March 2021 and DYSTRICT from November 2022 to December 2024, so no patient can appear in both. The largest set of mutually independent cohorts is therefore three — Lee-Mateus, DYSTRICT and McNierney — and no outcome in this literature can exceed three independent cohorts. Fernandez-Bussy 2024 and 2025 are each chained to both Lee-Mateus and DYSTRICT and can never enter a pool containing either; where they report an outcome the independent set does not, they are described narratively. Pooled analyses below therefore use three cohorts wherever all three report a harmonizable endpoint and two where they do not.

### Risk of bias

All five studies were judged at serious overall risk of bias on ROBINS-I, driven chiefly by confounding by indication: procedure allocation was non-random and influenced by lesion size, pleural distance, bronchus sign, and local availability, and residual imbalance persisted despite propensity matching (for example, a higher prevalence of prior cancer and shorter pleural distance in the CTTB arm of DYSTRICT). The cancer-only pathway study [39] was additionally judged at critical risk for the diagnostic-yield question because participants were selected around a final malignant diagnosis. Domain-level judgements and rationales are \provided in Table 3.

**Table 3:** Risk-of-bias assessment (ROBINS-I) of the included non-randomized studies

Study	Design/tool	D1	D2	D3	D4	D5	D6	D7	Overall
Lee-Mateus 2023	Retrospective cohort/ROBINS-I	Serious	Moderate	Low	Low	Moderate	Serious	Moderate	SERIOUS
Fernandez-Bussy 2024	Retrospective cohort (subsolid)/ROBINS-I	Serious	Serious	Low	Low	Moderate	Serious	Moderate	SERIOUS
Fernandez-Bussy 2025	Retrospective cohort (cancer pathway)/ROBINS-I	Serious	Critical	Low	Low	Moderate	Serious	Serious	CRITICAL (for DY question)
DYSTRICT 2026	Retrospective cohort, 1:1 PSM/ROBINS-I	Serious	Moderate	Low	Low	Low	Low	Moderate	SERIOUS
McNierney 2026	Retrospective observational + genetic PSM/ROBINS-I	Serious	Moderate	Low	Low	Low	Low	Moderate	SERIOUS

Domains — D1 confounding; D2 selection of participants; D3 classification of interventions; D4 deviations from intended interventions; D5 missing data; D6 measurement of outcomes; D7 selection of the reported result. Judgements: Low/Moderate/Serious/Critical. RoB 2 is not applicable (no randomized trials)

**1. Lee-Mateus 2023**: No matching; the arms differ significantly in prior-cancer history (47.8% vs. 68.8%, *p* = 0.003) and smoking distribution (*p* = 0.03), whereas sex was balanced (50.4% vs. 50.9%, *p* = 1.0) and pleural distance differed non-significantly (1.5 vs. 1.3 cm, *p* = 0.065). Intermediate/accuracy DY definition with follow-up adjudication inflates yield vs. strict endpoint (outcome-measurement bias)

**2. Fernandez-Bussy 2024**: Very small (*n* = 65); PS-weighting only; significant nodule-location imbalance; intermediate DY definition; subsolid-only limits applicability; wide imprecision

**3. Fernandez-Bussy 2025**: Participants selected on/around a cancer diagnosis (staging pathway) -> selection into the study is related to outcome; malignancy-only yield, no benign denominator. Appropriate only as narrative pathway evidence

**4. DYSTRICT 2026**: Strict ATS/ACCP DY (low outcome-measurement bias) & PSM improve validity, but residual confounding: cancer history 86% (CTTB) vs. 59% (ssRAB) and shorter pleural distance in CTTB not fully balanced; lesion-level unit (within-patient clustering)

**5. McNierney 2026**: Strict DY; good post-match SMD balance (< 0.10). Primary DY reported on unmatched 489 vs. 149 (confounding by indication); matching-with-replacement reuses controls; single center. Objective outcomes reduce detection bias

### Primary outcome: strict diagnostic yield

**Fig. 2:**
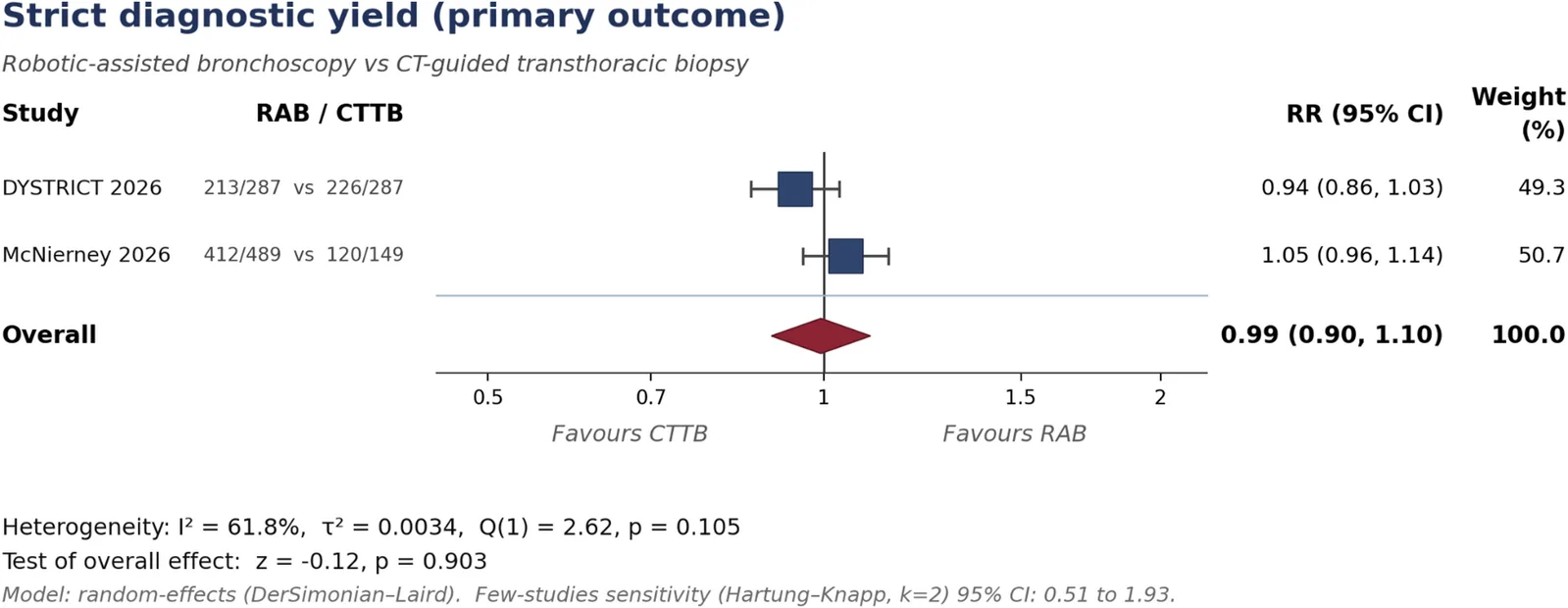
Forest plot of strict diagnostic yield (risk ratio, RAB versus CTTB) for the two independent cohorts that report the strict 2024 ATS/ACCP definition. Squares are study-specific risk ratios sized by random-effects weight, with 95% confidence intervals; the maroon diamond is the pooled DerSimonian–Laird estimate. Heterogeneity statistics, the test of overall effect and the few-studies Hartung–Knapp interval are shown beneath the plot. With only two cohorts the Hartung–Knapp interval (0.51–1.93) is uninformative

Only DYSTRICT and the McNierney cohort report strict ATS/ACCP yield; the other three reports do not permit a strict yield to be computed. In DYSTRICT, strict yield was 213/287 (74%) with RAB versus 226/287 (79%) with CTTB (*p* = 0.20) [40]; in the McNierney cohort it was 412/489 (84.3%) versus 120/149 (80.5%) (*p* = 0.35) [41]. The pooled RR was 0.99 (95% CI 0.90–1.10) by DerSimonian–Laird random effects, essentially identical under fixed-effect (0.99, 0.93–1.06) and Mantel–Haenszel (0.99, 0.93–1.05) models (Fig. 2). The two point estimates fall on opposite sides of the null (RR 0.94 versus 1.05), heterogeneity was nominally moderate (I²=62%, τ²=0.003), and the two-cohort HKSJ interval was 0.51–1.93. That interval spans both a halving and a doubling of yield, so this analysis is uninformative about equivalence and is reported for completeness rather than as an estimate of effect.

### Study-defined diagnostic yield across three independent cohorts

**Fig. 3:**
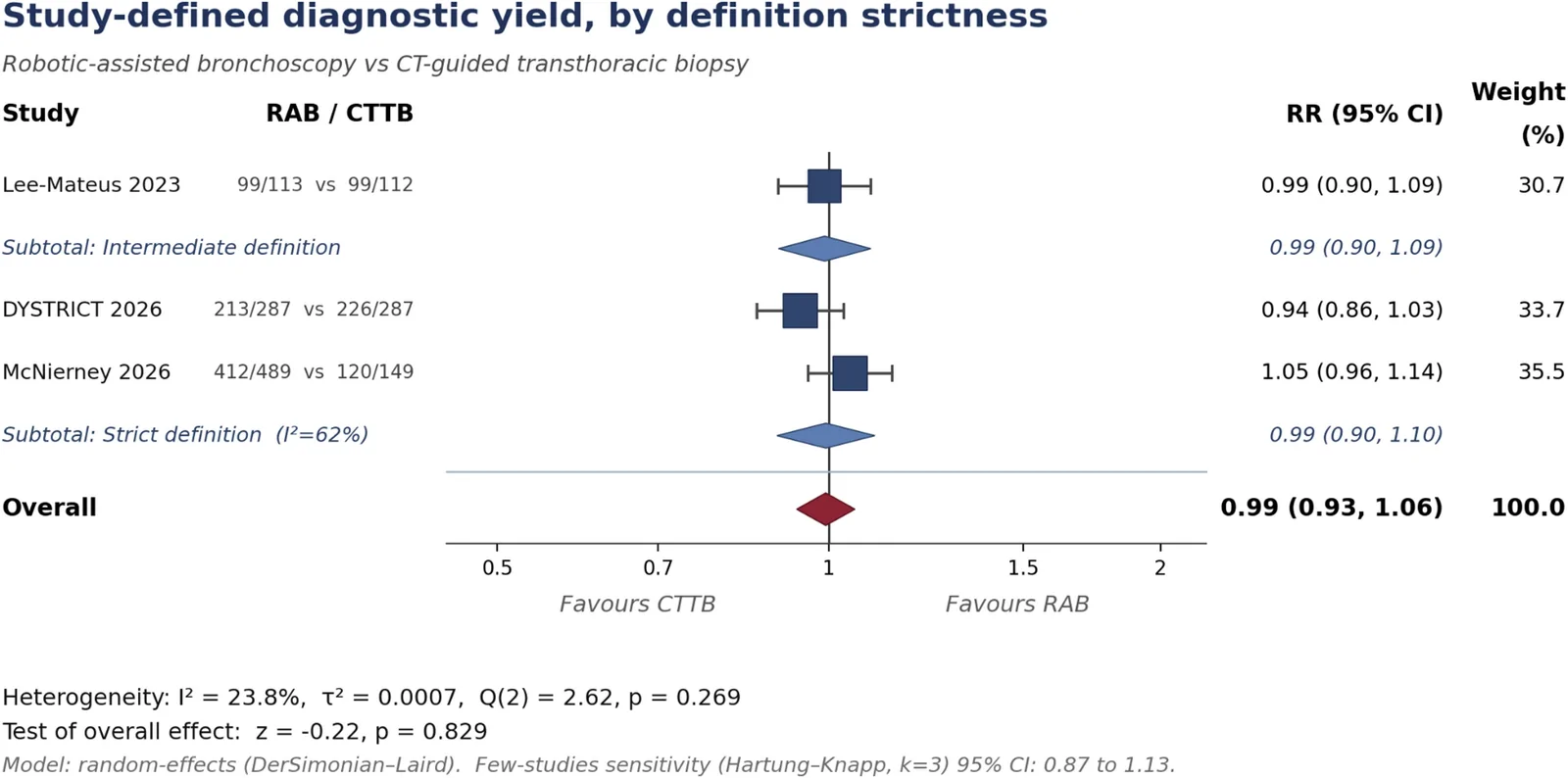
Forest plot of study-defined diagnostic yield across the three mutually independent cohorts, subgrouped by whether the study used a strict or an intermediate yield definition. Blue diamonds are subgroup random-effects estimates; the maroon diamond is the overall estimate. The relative effect is identical under both definitions even though absolute yields are lower under strict criteria. Plot conventions otherwise as in Fig. 2

Because the strict-definition pool is confined to two cohorts, we also pooled each study’s own yield definition across the full independent set of three (Fig. 3). Yields were 99/113 (87.6%) versus 99/112 (88.4%) in Lee-Mateus [37], 213/287 (74%) versus 226/287 (79%) in DYSTRICT [40] and 412/489 (84.3%) versus 120/149 (80.5%) in the McNierney cohort [41]. The pooled RR was 0.99 (95% CI 0.93–1.06) with low heterogeneity (I²=24%, τ²=0.0007, Q(2) = 2.62, *p* = 0.27) and a three-cohort HKSJ interval of 0.87–1.13, narrow enough to exclude any difference larger than about 13% in either direction. The subgroup analysis found an identical relative effect under the intermediate definition (RR 0.99, 95% CI 0.90–1.09) and the strict definition (RR 0.99, 95% CI 0.90–1.10), even though the absolute yields fell from 87.6% to 88.4% under the intermediate definition to 74–84% and 79–81% under strict criteria. Definitional strictness therefore lowers measured performance in both arms to a similar degree and does not change the comparison between them.

### Safety outcomes

**Fig. 4:**
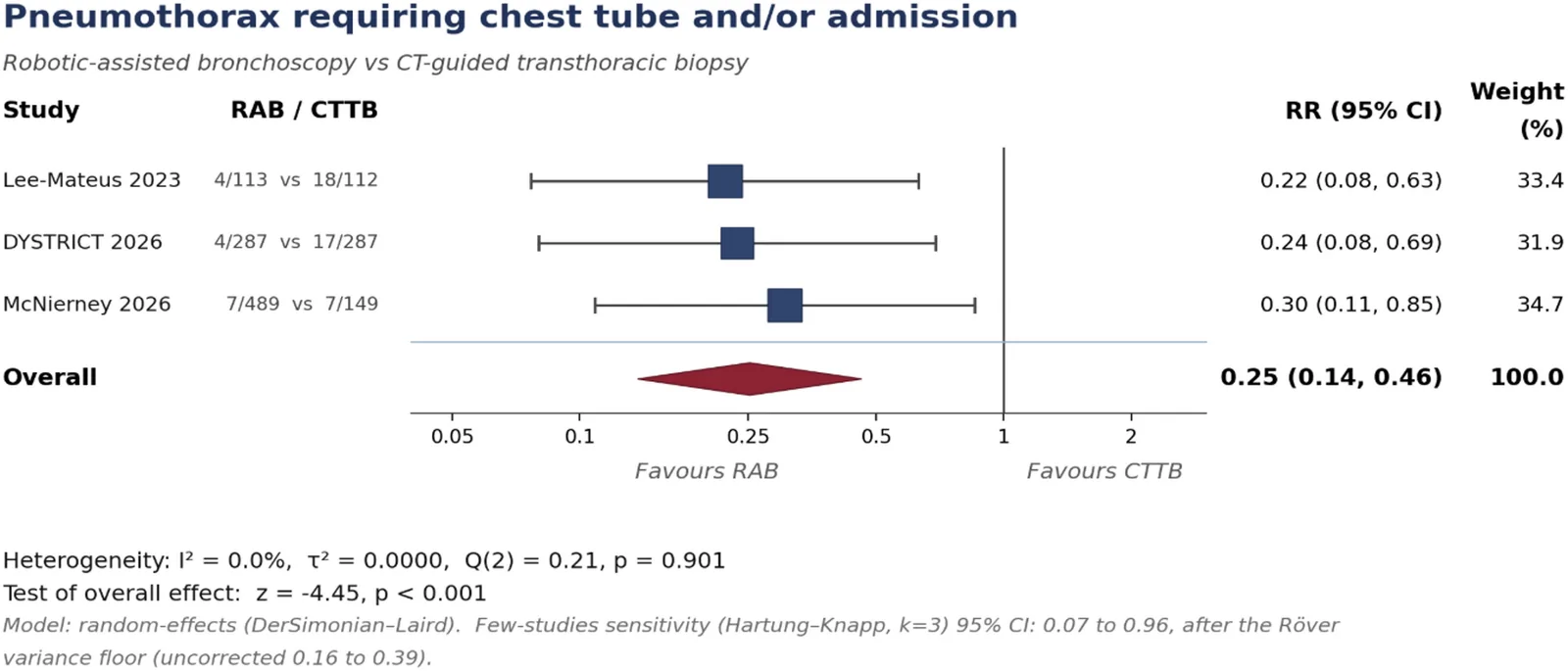
Forest plot of pneumothorax requiring a chest tube and/or hospital admission across the three mutually independent cohorts. Plot conventions as in Fig. 2. Endpoint wording differs slightly between cohorts (admission or chest tube in Lee-Mateus; chest tube alone in DYSTRICT; chest tube and admission in the McNierney cohort), yet the three estimates are closely concordant and the three-cohort Hartung–Knapp interval still excludes the null. This is the primary safety analysis of the review

**Fig. 5:**
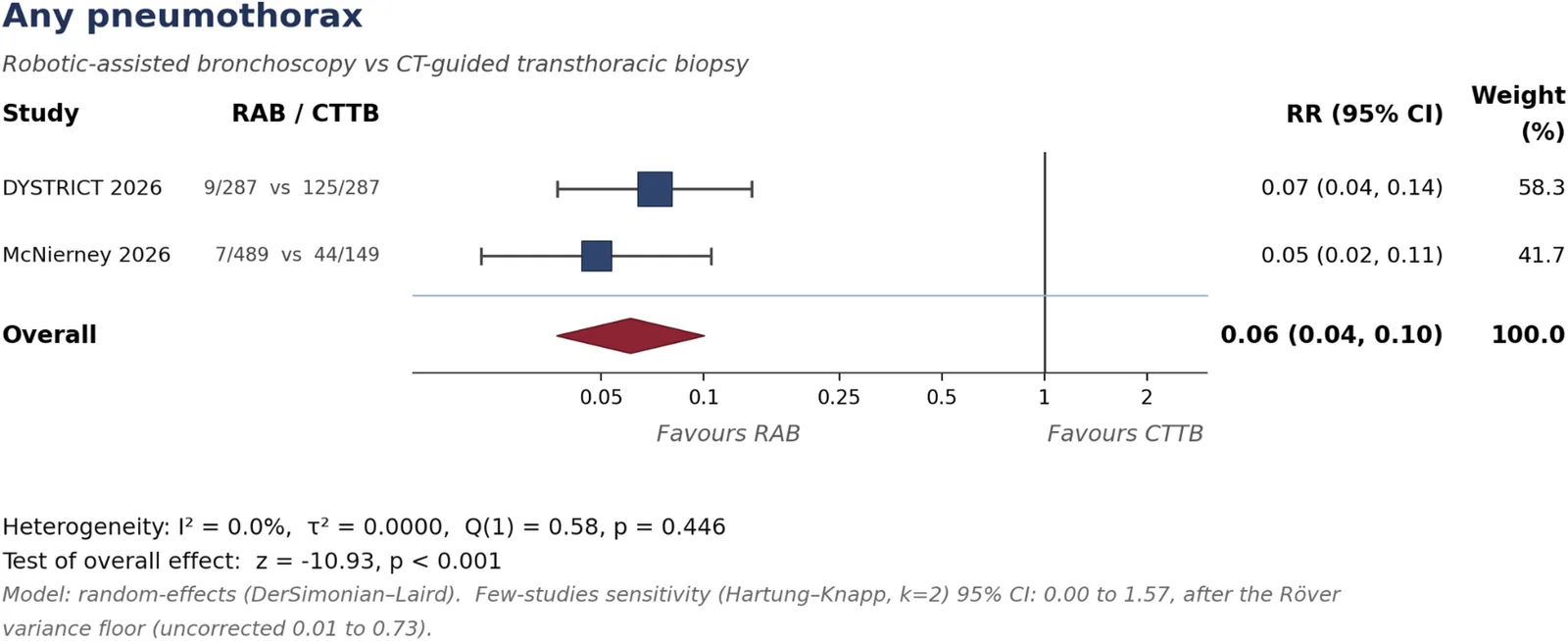
Forest plot of any radiographically detected pneumothorax (risk ratio, RAB versus CTTB) for the two cohorts reporting this endpoint. Plot conventions as in Fig. 2. A risk ratio below 1.0 favors RAB. Note that although the effect is very large and consistent, the two-cohort Hartung–Knapp interval crosses the null

Pneumothorax requiring a chest tube and/or hospital admission was reported by all three independent cohorts and is the most robust finding of this review: 4/113 (3.5%) versus 18/112 (16.1%) in Lee-Mateus [37], 4/287 (1.4%) versus 17/287 (5.9%) in DYSTRICT [40], and 7/489 (1.4%) versus 7/149 (4.7%) in the McNierney cohort [41]. The three study estimates were closely concordant (RR 0.22, 0.24 and 0.30) with no detectable heterogeneity (I²=0%, Q(2) = 0.21, *p* = 0.90), giving a pooled RR of 0.25 (95% CI 0.14–0.46) and a three-cohort HKSJ interval of 0.07–0.96 that still excludes the null (Fig. 4). The endpoint wording differs across the three reports — admission or chest tube in Lee-Mateus, chest tube alone in DYSTRICT, chest tube and admission in the McNierney cohort — so this is a composite, but the concordance of the estimates suggests those differences matter little in practice. Any radiographically detected pneumothorax, reported by two cohorts, showed a much larger effect (9/287 [3%] versus 125/287 [44%]; 7/489 [1.4%] versus 44/149 [29.5%]; pooled RR 0.06, 95% CI 0.04–0.10, I²=0%, absolute risk reduction approximately 34% points; Fig. 5), and restricting to chest-tube placement alone gave a pooled RR of 0.27 (95% CI 0.13–0.57; I²=0%). Both two-cohort estimates are highly significant under conventional models but neither survives the Rover-corrected HKSJ interval (0.00–1.57 and 0.00–33.68 respectively). That is a property of inference on one degree of freedom rather than of the data — with two cohorts the t multiplier is 12.7 — and it is the reason we base our conclusions about pleural complications on the three-cohort composite.

### Procedure duration

**Fig. 6:**
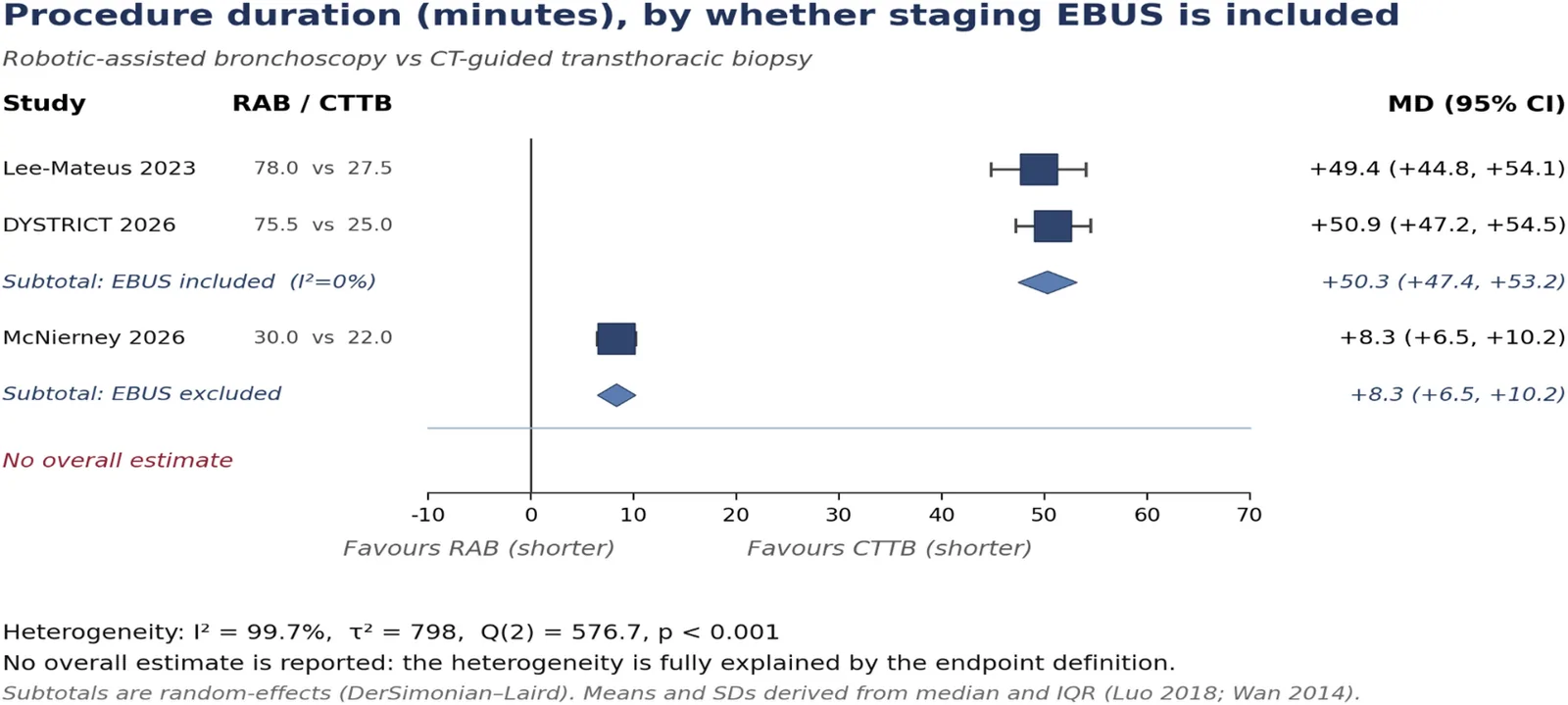
Forest plot of procedure duration (mean difference in minutes, RAB minus CTTB) across the three mutually independent cohorts, subgrouped by whether same-session staging endobronchial ultrasound is counted in the robotic time. Means and standard deviations were derived from reported medians and interquartile ranges. No overall estimate is shown because the heterogeneity (I²=99.7%) is entirely attributable to this definitional difference. A mean difference above zero indicates a longer RAB procedure

All three independent cohorts reported procedure duration, but not the same endpoint: Lee-Mateus and DYSTRICT include same-session staging endobronchial ultrasound in the robotic time, whereas the McNierney cohort measures robot insertion to removal only. All mean differences are expressed as RAB minus CTTB, so that a positive value denotes a longer robotic procedure. Derived mean differences were + 49.4 min (95% CI + 44.8 to + 54.1) and + 50.9 min (+ 47.2 to + 54.5) longer than CTTB in the two studies that include staging, and + 8.3 min (+ 6.5 to + 10.2) longer than CTTB in the study that excludes it. Overall heterogeneity was accordingly extreme (I²=99.7%, Q(2) = 576.7, *p* < 0.001) and we do not report an overall pooled estimate; the two subgroups were internally homogeneous (+ 50.3 min longer than CTTB, 95% CI + 47.4 to + 53.2, I²=0%, where staging is included; +8.3 min longer where it is not) (Fig. 6). The heterogeneity is thus fully explained by the endpoint definition, and that is itself the finding: most of the apparent time penalty of RAB is the mediastinal staging performed in the same anesthetic event, not the nodule biopsy.

### Secondary and pathway outcomes (narrative synthesis)

**Fig. 7:**
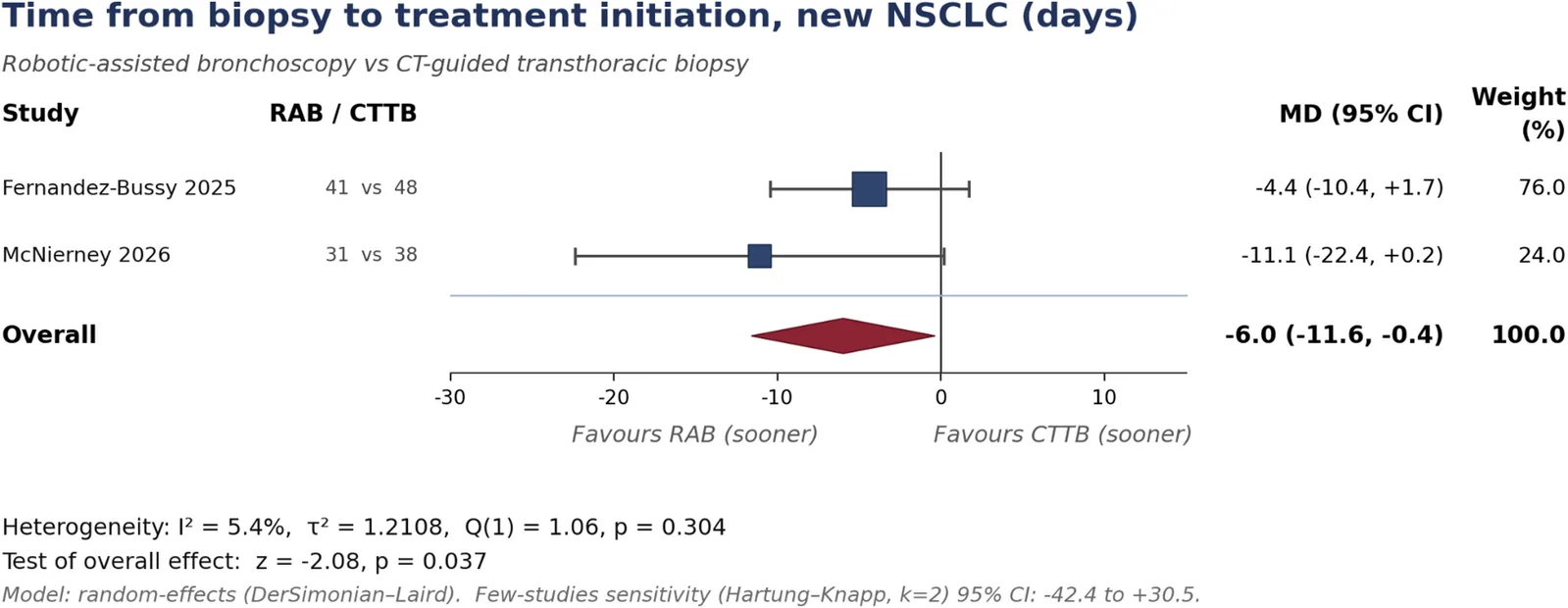
Forest plot of time from biopsy to treatment initiation for new non–small-cell lung cancer (mean difference in days, RAB minus CTTB) for the two mutually independent cohorts reporting it. Plot conventions as in Fig. 6. A mean difference below zero indicates earlier treatment after RAB. Exploratory: neither crude comparison was individually significant and the Hartung–Knapp interval crosses the null

Time from biopsy to treatment initiation for new non–small-cell lung cancer was reported by two mutually independent cohorts at different campuses: median 41 versus 48 days (*p* = 0.297) in the pathway report [39] and 31 versus 38 days (*p* = 0.098) in the McNierney cohort [41]. Neither crude comparison was significant; the pooled mean difference was − 6.0 days (95% CI − 11.6 to − 0.4; I²=5%) but the HKSJ interval crossed the null (− 42.4 to + 30.5), and the pathway report does not state its index date, so this analysis is exploratory (Fig. 7). The adjusted McNierney estimate (− 22.3 days) is also three times its crude median difference. The remaining outcomes were reported by a single independent cohort and are described narratively. Hospital admission for a complication was lower with RAB in DYSTRICT (9/287 [3%] versus 24/287 [8%]; odds ratio [OR] 0.35, 95% CI 0.15–0.75) [40] and directionally concordant in the overlapping pathway report (5.4% versus 19.6%) [39]. Molecular/genomic adequacy favored RAB in the McNierney cohort (206/230 [89.6%] versus 72/91 [79.1%]; adjusted OR 2.21, 95% CI 1.18–4.16) [41]. Pulmonary hemorrhage was rare with RAB and far more frequent with CTTB (1/489 [0.2%] versus 21/149 [14.1%]; adjusted OR 0.02, 95% CI 0.003–0.17) [41], while DYSTRICT reported negligible bleeding volumes in both arms [40]. RAB was associated with more early-stage diagnoses in the pathway report (114/148 [77.0%] versus 79/148 [53.4%]; OR 3.02, 95% CI 1.83–5.04) [39]. Same-session mediastinal staging was achieved in 92/113 (81.4%) of RAB procedures in Lee-Mateus and 280/287 (98%) in DYSTRICT, versus 42/112 (37.5%) and 269/287 of CTTB patients respectively who required a separate staging procedure [37, 40]; the denominators differ too much to pool. DYSTRICT also found fewer secondary procedures to re-assess the nodule after RAB (18/287 [6%] versus 31/287 [11%]; *p* = 0.052) [40]. Procedure-related mortality was sought and was not reported by any study; no study provided an explicit zero-event statement. Two cohorts related diagnostic yield to lesion size. DYSTRICT reported strict yield by size category within each arm—65% (< 10 mm), 76% (10–20 mm) and 77% (> 20 mm) with RAB versus 80%, 74% and 83% with CTTB, with no significant within-arm trend (*p* = 0.180 and *p* = 0.241)—but did not report the number of lesions in each stratum, so no stratum-specific risk ratio or confidence interval can be derived [40]. The McNierney cohort modelled yield against nodule size as a continuous restricted cubic spline stratified by procedure and reported that yield rose with size to approximately 30 mm and then plateaued in both arms, without tabulated strata [41]. Location was reported only at lobar level: within the RAB arm of DYSTRICT, yield was lower for lower-lobe than upper-lobe lesions (odds ratio 0.60, 95% CI 0.35–1.01, *p* = 0.061) [40]. No study reported yield or complications according to the presence or absence of a bronchus sign—the McNierney cohort used bronchus sign only as a matching covariate—and none reported results by lung zone or for apical, retrocostal or outer-third lesions. A quantitative subgroup analysis by lesion size or accessibility was therefore not possible.

### Certainty of evidence

Certainty was rated for each pooled outcome (Table 4). It was low for pneumothorax requiring intervention, where serious risk of bias and indirectness were offset by a large, entirely consistent effect that persists under the few-studies correction, and low for the staging-inclusive procedure-duration subgroup. It was very low for strict diagnostic yield (serious risk of bias, indirectness, inconsistency and critical imprecision), for study-defined yield (serious risk of bias and indirectness, despite good consistency and precision), for any pneumothorax and for chest-tube placement (two cohorts each, neither robust to the few-studies correction), and for time to treatment, hospital admission and molecular adequacy (exploratory two-cohort or single-cohort estimates).

**Table 4:** Summary of findings (GRADE)

Outcome	Pooled effect (RAB vs. CTTB)	Cohorts/*N*	Certainty	Reasons for rating
Pneumothorax requiring chest tube and/or admission	RR 0.25 (0.14–0.46)	3/1,437	⨁⨁◯◯** Low**	Serious risk of bias and indirectness, offset by a large, entirely consistent effect (I² = 0%) that persists under the few-studies correction (HKSJ 0.07–0.96). Composite endpoint wording differs across cohorts
Study-defined diagnostic yield	RR 0.99 (0.93–1.06)	3/1,437	⨁◯◯◯** Very low**	Serious risk of bias; indirectness (single health system). Consistency (I² = 24%) and precision (HKSJ 0.87–1.13) are good, and the relative effect is identical under strict and intermediate definitions
Strict diagnostic yield (primary outcome)	RR 0.99 (0.90–1.10)	2/1,212	⨁◯◯◯ **Very low**	Serious risk of bias; indirectness; inconsistency (I² = 62%, opposing point estimates); critical imprecision (HKSJ 0.51–1.93 spans halving to doubling)
Any pneumothorax	RR 0.06 (0.04–0.10)	2/1,212	⨁◯◯◯** Very low**	Serious risk of bias and indirectness; very large and consistent effect (I²=0%) but only two cohorts, and the estimate is not robust to the few-studies correction (HKSJ 0.00–1.57)
Chest-tube placement alone	RR 0.27 (0.13–0.57)	2/1,212	⨁◯◯◯** Very low**	Serious risk of bias and indirectness; serious imprecision (HKSJ 0.00–33.68) and pooling of non-identical endpoints. Superseded by the three-cohort composite above
Procedure duration, staging included	MD + 50.3 min (+ 47.4 to + 53.2)	2/799	⨁⨁◯◯** Low**	Serious risk of bias and indirectness, offset by a very large, entirely consistent effect (I² = 0%). Means derived from medians and IQRs
Procedure duration, staging excluded	MD + 8.3 min (+ 6.5 to + 10.2)	1/638	⨁◯◯◯** Very low**	Single cohort; serious risk of bias; different endpoint definition from the cohorts above
Time to treatment, new NSCLC	MD − 6.0 days (− 11.6 to − 0.4)	2/411	⨁◯◯◯** Very low**	Both crude comparisons non-significant; only the pool reaches nominal significance and the HKSJ interval crosses the null; index date not stated in one cohort
Hospital admission	OR 0.35 (0.15–0.75)	1/574	⨁◯◯◯** Very low**	Single cohort; serious risk of bias; imprecision
Molecular adequacy	aOR 2.21 (1.18–4.16)	1/321	⨁◯◯◯** Very low**	Single cohort; serious risk of bias; denominator restricted to the malignant/tested subset (230 RAB, 91 CTTB)

aOR, adjusted odds ratio; CI, confidence interval; HKSJ, Hartung–Knapp–Sidik–Jonkman; IQR, interquartile range; MD, mean difference; NSCLC, non–small-cell lung cancer; OR, odds ratio; RR, risk ratio. Cohorts are mutually independent; N is the sum of both arms of all contributing cohorts. All pooled estimates are DerSimonian–Laird random effects. HKSJ intervals include the Rover ad-hoc variance floor. GRADE certainty: ⨁⨁⨁⨁ high; ⨁⨁⨁◯ moderate; ⨁⨁◯◯ low; ⨁◯◯◯ very low

## Discussion

In this first systematic review restricted to direct RAB-versus-CTTB comparisons, four findings emerge. First, diagnostic yield is comparable. Across all three mutually independent cohorts the pooled RR was 0.99 (95% CI 0.93–1.06) with a Hartung–Knapp interval of 0.87–1.13, which excludes any difference larger than about 13% in either direction; the two-cohort strict-definition pool gave the same point estimate but an uninformative interval (0.51–1.93). Second, RAB was associated with a large, consistent and biologically coherent reduction in pleural complications. Pneumothorax requiring a chest tube and/or admission was about three-quarters lower with RAB (RR 0.25, 95% CI 0.14–0.46) in an analysis drawing on all three independent cohorts that shows no heterogeneity at all and remains significant under the conservative few-studies correction (0.07–0.96). The two-cohort estimates for any pneumothorax (RR 0.06) and chest tube alone (RR 0.27) are far larger but, resting on a single degree of freedom, do not survive that correction, so we treat the three-cohort composite as the primary safety result. Third, most of the procedure-time penalty attributed to RAB is staging rather than biopsy: RAB took about 50 min longer than CTTB where same-session mediastinal staging was counted in the robotic time but only 8 min longer where it was not, and that single distinction accounts for essentially all of the between-study heterogeneity in duration. Fourth, RAB was associated with a series of downstream differences — fewer hospital admissions, higher molecular adequacy, earlier treatment and near-universal same-session mediastinal staging — although these single-cohort or exploratory observational associations may partly reflect selection by lesion characteristics, center expertise and staging availability rather than an effect of RAB itself. Every included study was retrospective and drawn from a single health system, so certainty remains low to very low and these conclusions are hypothesis-generating.

Our findings add to the existing comparative literature. The only prior quantitative comparison was an ERS network meta-analysis that ranked five biopsy modalities but derived the RAB-versus-CT contrast almost entirely from indirect evidence, because only a single direct study existed at the time [22]. A separate 2026 systematic review compared robotic needle-guidance systems with conventional CT-guided percutaneous biopsy [42]; despite a closely similar title, that review evaluates a robotic percutaneous platform, includes no bronchoscopic arm, and therefore does not address the transbronchial-versus-transthoracic contrast examined here. By restricting to head-to-head cohorts published through 2026 and to a uniform strict definition, we provide the first direct pooled estimate of comparative yield. Our result also sits alongside two contemporary randomized trials: RAB was superior to electromagnetic navigation bronchoscopy in RELIANT [24], and navigational bronchoscopy was noninferior to transthoracic needle biopsy in VERITAS [23]. Taken together these trials provide indirect context suggesting broadly similar diagnostic performance between the bronchoscopic and percutaneous routes; transitivity is nevertheless uncertain, because VERITAS adjudicated a 12-month, follow-up-confirmed accuracy endpoint rather than strict index-procedure yield, so the assumptions linking the two trials are definitional as well as structural, and they do not establish non-inferiority of RAB to CTTB. Our direct observational data are consistent with no statistically detectable difference between the two modalities, but likewise do not establish equivalence or non-inferiority.

The absolute RAB yields we observed (74% and 84.3% under the strict definition) sit at or above the pooled estimates from modality-specific RAB meta-analyses, which report 84.3% and 80.4% overall [17, 18] and, when yield definition is separated out, 86.6% (95% CI 83.7–89.2) under intermediate criteria but only 69.6% (95% CI 61.8–76.8) under strict criteria [19]. DYSTRICT’s 74% falls within that strict-criteria interval; the McNierney cohort’s 84.3% lies above it, which is consistent with a high-volume centre using cone-beam CT, rapid on-site evaluation and cryobiopsy. Both figures represent a substantial advance over the historical performance of guided bronchoscopy, whose pooled yield was 70.5% before 2012 and 69.2% afterwards despite a decade of technological change [9, 10], and over real-world registries such as AQuIRE, where yield ranged from 38.5% to 63.7% depending on the adjunct used [11]. Critically, the apparent diagnostic superiority of CTTB seen in older, definition-heterogeneous comparisons narrowed to non-significance once nonspecific and follow-up-informed results were excluded. This shows how permissive yield definitions inflate measured performance, and supports the ATS/ACCP framework [20, 21].

The safety advantage of the bronchoscopic route is the most robust signal in our analysis and is strongly corroborated externally. Meta-analytic data place the pneumothorax risk of transthoracic biopsy at 18.8% after fine-needle aspiration and 25.3% after core biopsy, with chest-tube placement in 4.3% and 5.6% respectively [7, 8]. The randomized VERITAS trial reported an almost identical gradient (pneumothorax 3.3% with navigational bronchoscopy versus 28.3% with transthoracic biopsy) and, importantly for the present analysis, an endpoint that is definitionally close to our three-cohort composite: pneumothorax requiring chest-tube placement or hospitalisation occurred in 0.8% versus 11.5% of patients [23]. That randomized contrast is of the same direction and larger magnitude than our pooled risk ratio of 0.25, so if anything our observational estimate is conservative. The higher CTTB pneumothorax rates in our included cohorts (30–44%) probably reflect the counting of both symptomatic and asymptomatic events and the referral of deeper, more challenging lesions to a tertiary centre; nonetheless, the direction and magnitude of the effect are consistent across cohorts and expected on mechanistic grounds, because the transbronchial route does not violate the visceral pleura. Judged against the Bradford Hill considerations — effect size, consistency, biological plausibility and coherence — residual confounding alone is unlikely to account for a difference of this magnitude, although it cannot be formally excluded; this is why the three-cohort pleural-intervention outcome retains low rather than very low certainty despite its observational origins. The two-cohort any-pneumothorax estimate is rated very low, not because the effect is doubtful but because two cohorts cannot support precise inference.

Two further advantages deserve attention. First, RAB was associated with higher molecular adequacy (89.6% versus 79.1%) [41]. Our single-cohort finding should not be over-read, but it is consistent with a 2025 American Association of Bronchology and Interventional Pulmonology/International Association for the Study of Lung Cancer clinical practice guideline, which concluded after a modified Delphi process that guided-bronchoscopy specimens are of comparable adequacy to percutaneous specimens for comprehensive biomarker testing while carrying a materially better safety profile [43]. That guideline provides the external, multidisciplinary corroboration that a single retrospective cohort cannot. Mechanistically, larger and better-preserved cryobiopsy cores are the most plausible explanation: in shape-sensing RAB, cryobiopsy has been reported to raise diagnostic yield substantially over fine-needle aspiration and to deliver specimens adequate for molecular analysis in essentially all cases, and both cohorts contributing to our safety analyses used a 1.1 mm cryoprobe [37, 40, 41]. Selection and centre effects nevertheless cannot be excluded. Second, the ability to biopsy the lesion and stage the mediastinum in a single anesthetic event was associated with a higher proportion of early-stage diagnoses and, on exploratory two-cohort evidence, with earlier treatment [39, 41] — outcomes with direct prognostic relevance. These pathway efficiencies, rather than diagnostic yield in isolation, may prove to be the more important differentiators between the two strategies.

A pooled average across general cohorts may also understate where the two modalities genuinely differ. The distinctive capability of shape-sensing robotic platforms is the ability to reach lesions in anatomically demanding territory — the extreme outer third of the parenchyma, apical and retrocostal segments, and lesions whose percutaneous trajectory would traverse a long segment of aerated or emphysematous lung — and to sample sub-centimetre targets in which respiratory motion, target displacement and catheter stability become the decisive determinants of success. Effect modification is plausible in both directions: the pleural-safety advantage of the transbronchial route should widen as pleural distance and the number of pleural passes increase, whereas CTTB is likely to retain a yield advantage for lesions that lack a bronchus sign or lie beyond bronchoscopic reach. The data needed to test either proposition are, however, almost entirely missing, and the little that exists does not favour the robotic platform in the smallest lesions. DYSTRICT is the only cohort to report strict yield by size category in both arms, and below 10 mm its yield was numerically lower with RAB than with CTTB (65% versus 80%), with the 10–20 mm and > 20 mm strata favouring neither modality; none of these trends was significant and no stratum denominators were published, so no effect estimate can be attached to them [40]. The McNierney spline analysis shows yield rising with nodule size to approximately 30 mm and then plateauing in both arms in parallel, again without tabulated strata [41]. No study reports yield or complications by bronchus sign or by lung zone at all. The constant relative effect we observed is therefore an average over a lesion mix that is itself a product of referral practice, and the one cohort that disaggregates by size gives no indication that robotic bronchoscopy outperforms CTTB in sub-centimetre targets — although those strata are small, unadjusted and drawn from a single centre, and cannot exclude a real advantage either. Prespecified stratification by lesion size, bronchus sign, pleural depth and lung zone, reported with denominators rather than percentages alone, is what future comparative studies, meta-analyses and prospective registries must supply, because it is precisely in these subgroups that the two strategies are least interchangeable.

The procedure-time finding deserves a further qualification. The roughly 50-minute difference we observed where staging was bundled into the robotic time is not a fixed property of the technology: in a cumulative-sum analysis of nine proceduralists at a high-volume centre, median single-target RAB time fell from 62 min across the first ten cases to 39 min after case 40, with the competency threshold crossed at about the 25th lesion [44]. Two of our three independent cohorts recruited from the earliest years of their institutional robotic programme, so part of the measured time penalty is likely to be learning curve rather than steady-state practice. The same consideration cuts the other way for diagnostic yield and complications, where early-experience cases would if anything bias against RAB. For clinical practice, these data suggest that, in centres with robotic capability, RAB offers diagnostic performance comparable to that of CTTB with a more favorable pleural-safety profile and greater same-session staging and molecular efficiency, although the last two observations rest on single-cohort data. The comparative advantage is likely to be greatest for patients at elevated risk of pneumothorax — those with emphysema, prior pneumonectomy, bullous disease, or lesions requiring traversal of aerated lung — and for those in whom same-session nodal staging or ample tissue for genomic profiling is a priority. Conversely, CTTB retains important advantages in accessibility, procedural speed, lower capital cost, and the ability to reach lesions that lack a bronchus sign or lie beyond bronchoscopic range. The decision is therefore best framed as individualized, resource-aware shared decision-making rather than the wholesale replacement of one modality by the other.

The strengths of this review are methodological. To our knowledge it is the first synthesis restricted to direct RAB-versus-CTTB comparisons; its cohort-genealogy step prevents the patient double-counting that is an under-recognized hazard in an institution-dominated literature, and by identifying the largest mutually independent set of cohorts for each outcome separately it extracts three-cohort estimates from a literature that a naive reading would reduce to two; and it applies few-studies-appropriate statistics (Hartung–Knapp with the Rover variance floor, and Mantel–Haenszel) alongside transparent GRADE rating, so that a small evidence base is not over-interpreted.

Several limitations must temper interpretation. The entire eligible evidence base is retrospective and originates from a single, high-volume, expert health system; no independent centre has yet published a direct comparison, so both external validity and the influence of operator learning curves remain uncertain. At most three mutually independent cohorts contributed to any outcome, and only two to strict diagnostic yield, any pneumothorax and chest-tube placement; for those three outcomes the few-studies Hartung–Knapp interval is uninformative, and we have accordingly based our conclusions on the three-cohort analyses. Allocation was non-random and confounding by indication persisted despite propensity matching. Heterogeneity in outcome definitions, in the unit of analysis (patient versus lesion) and in composite endpoints constrained pooling; the pleural-intervention outcome in particular combines three similar but not identical endpoint definitions. Stratified data on lesion difficulty are almost absent: only one cohort reported yield by lesion size category and it did not report the denominators of those strata, no study reported yield by bronchus sign or by lung zone, and no study reported complications by lesion size. We therefore could not quantify the subgroups in which the two modalities are least likely to be interchangeable, and our pooled estimates are averages over the lesion mix referred to a tertiary centre that should not be assumed to hold for every lesion phenotype, in particular for sub-centimetre or bronchoscopically inaccessible targets. Small-study and publication-bias assessment was not feasible with fewer than ten cohorts. Embase and Cochrane CENTRAL, both prespecified in the protocol, were not searched and Europe PMC was substituted; although citation searching identified no eligible study beyond the five included, we cannot exclude the possibility that an unpublished conference abstract was missed. Two of the five reports also contain internally inconsistent figures, which we have tabulated rather than silently resolved. Finally, none of the studies reported cost, quality-of-life or long-term survival outcomes, leaving the full value proposition of each modality incompletely characterized.

An adequately powered, multicentre randomized trial comparing RAB directly with CTTB—using a prespecified strict yield definition, independent and blinded outcome adjudication, and co-primary endpoints of diagnostic yield (noninferiority) and pleural complications (superiority)—is the necessary next step, and the concordant safety signal we report provides both the rationale and the equipoise for it. Such a trial should be complemented by prespecified subgroup analyses (lesion size, including ≤ 10 mm and 10–20 mm strata reported with their denominators; subsolid morphology; bronchus sign; pleural distance; and lesion location, including apical, retrocostal and outer-third lesions), formal health-economic evaluation, learning-curve characterization, and patient-reported outcomes, so that the comparative value of these technologies can ultimately be judged by effectiveness, safety, and cost.

## Conclusions

Low-certainty, single–health–system evidence indicates that robotic-assisted bronchoscopy is associated with substantially lower rates of pneumothorax and chest-tube placement, and with higher molecular adequacy and same-session staging, while showing no statistically detectable difference in diagnostic yield versus CT-guided transthoracic biopsy (equivalence not established). Because stratified results are available from a single cohort for lesion size and from none for bronchus sign or lung zone, the relative performance of the two modalities in the smallest and least accessible lesions remains undefined. These hypothesis-generating findings provide a strong rationale for a multicentre randomized trial and for incorporating the lower risk of pleural complications with the robotic approach into shared decision-making.

## Supplementary Information

Below is the link to the electronic supplementary material.

Supplementary Material 1

## Data Availability

No datasets were generated or analysed during the current study.

## References

[1] Siegel RL, Kratzer TB, Giaquinto AN, Sung H, Jemal A (2025) Cancer statistics, 2025. CA Cancer J Clin 75(1):10–45. 10.3322/caac.21871 39817679 PMC11745215

[2] National Lung Screening Trial Research Team, Aberle DR, Adams AM, Berg CD et al (2011) Reduced lung-cancer mortality with low-dose computed tomographic screening. N Engl J Med 365(5):395–409. 10.1056/NEJMoa1102873 21714641 PMC4356534

[3] de Koning HJ, van der Aalst CM, de Jong PA et al (2020) Reduced lung-cancer mortality with volume CT screening in a randomized trial. N Engl J Med 382(6):503–513. 10.1056/NEJMoa1911793 31995683

[4] MacMahon H, Naidich DP, Goo JM et al (2017) Guidelines for management of incidental pulmonary nodules detected on CT images: from the Fleischner Society 2017. Radiology 284(1):228–243. 10.1148/radiol.2017161659 28240562

[5] Gould MK, Donington J, Lynch WR et al (2013) Evaluation of individuals with pulmonary nodules: when is it lung cancer? Diagnosis and management of lung cancer, 3rd ed: American College of Chest Physicians evidence-based clinical practice guidelines. Chest 143(5 Suppl):e93S–e120S. 10.1378/chest.12-2351 23649456 PMC3749714

[6] Mazzone PJ, Lam L (2022) Evaluating the patient with a pulmonary nodule: a review. JAMA 327(3):264–273. 10.1001/jama.2021.24287 35040882

[7] Heerink WJ, de Bock GH, de Jonge GJ, Groen HJM, Vliegenthart R, Oudkerk M (2017) Complication rates of CT-guided transthoracic lung biopsy: meta-analysis. Eur Radiol 27(1):138–148. 10.1007/s00330-016-4357-8 27108299 PMC5127875

[8] Wiener RS, Schwartz LM, Woloshin S, Welch HG (2011) Population-based risk for complications after transthoracic needle lung biopsy of a pulmonary nodule: an analysis of discharge records. Ann Intern Med 155(3):137–144. 10.7326/0003-4819-155-3-201108020-00003 21810706 PMC3150964

[9] Wang Memoli JS, Nietert PJ, Silvestri GA (2012) Meta-analysis of guided bronchoscopy for the evaluation of the pulmonary nodule. Chest 142(2):385–393. 10.1378/chest.11-1764 21980059 PMC3425336

[10] Nadig TR, Thomas N, Nietert PJ et al (2023) Guided bronchoscopy for the evaluation of pulmonary lesions: an updated meta-analysis. Chest 163(6):1589–1598. 10.1016/j.chest.2022.12.044 36640994 PMC10925546

[11] Ost DE, Ernst A, Lei X et al (2016) Diagnostic yield and complications of bronchoscopy for peripheral lung lesions. Results of the AQuIRE registry. Am J Respir Crit Care Med 193(1):68–77. 10.1164/rccm.201507-1332OC 26367186 PMC4731617

[12] Folch EE, Pritchett MA, Nead MA et al (2019) Electromagnetic navigation bronchoscopy for peripheral pulmonary lesions: one-year results of the prospective, multicenter NAVIGATE study. J Thorac Oncol 14(3):445–458. 10.1016/j.jtho.2018.11.013 30476574

[13] Fielding DIK, Bashirzadeh F, Son JH et al (2019) First human use of a new robotic-assisted fiber optic sensing navigation system for small peripheral pulmonary nodules. Respiration 98(2):142–150. 10.1159/000498951 31352444

[14] Rojas-Solano JR, Ugalde-Gamboa L, Machuzak M (2018) Robotic bronchoscopy for diagnosis of suspected lung cancer: a feasibility study. J Bronchol Interv Pulmonol 25(3):168–175. 10.1097/LBR.0000000000000499 PMC6082666 29762461

[15] Chaddha U, Kovacs SP, Manley C et al (2019) Robot-assisted bronchoscopy for pulmonary lesion diagnosis: results from the initial multicenter experience. BMC Pulm Med 19(1):243. 10.1186/s12890-019-1010-8 31829148 PMC6907137

[16] Kalchiem-Dekel O, Connolly JG, Lin IH et al (2022) Shape-sensing robotic-assisted bronchoscopy in the diagnosis of pulmonary parenchymal lesions. Chest 161(2):572–582. 10.1016/j.chest.2021.07.2169 34384789 PMC8941601

[17] Ali MS, Ghori UK, Wayne MT, Shostak E, De Cardenas J (2023) Diagnostic performance and safety profile of robotic-assisted bronchoscopy: a systematic review and meta-analysis. Ann Am Thorac Soc 20(12):1801–1812. 10.1513/AnnalsATS.202301-075OC 37769170

[18] Zhang C, Xie F, Li R, Cui N, Herth FJF, Sun J (2024) Robotic-assisted bronchoscopy for the diagnosis of peripheral pulmonary lesions: a systematic review and meta-analysis. Thorac Cancer 15(7):505–512. 10.1111/1759-7714.15229 38286133 PMC10912532

[19] Li X, Bai J, Zhou X, Wang T, Zhang Y, Hu Y (2025) Diagnostic performance and safety for robotic-assisted bronchoscopy in pulmonary nodules: a systematic review and meta-analysis. Int J Surg 111(6):4020–4032. 10.1097/JS9.0000000000002423 40358662 PMC12165526

[20] Gonzalez AV, Silvestri GA, Korevaar DA et al (2024) Assessment of advanced diagnostic bronchoscopy outcomes for peripheral lung lesions: a Delphi consensus definition of diagnostic yield and recommendations for patient-centered study designs. An official American Thoracic Society/American College of Chest Physicians research statement. Am J Respir Crit Care Med 209(6):634–646. 10.1164/rccm.202401-0192ST 38394646 PMC10945060

[21] Vachani A, Maldonado F, Laxmanan B, Kalsekar I, Murgu S (2022) The impact of alternative approaches to diagnostic yield calculation in studies of bronchoscopy. Chest 161(5):1426–1428. 10.1016/j.chest.2021.08.074 34506792

[22] Balasubramanian P, Abia-Trujillo D, Barrios-Ruiz A et al (2024) Diagnostic yield and safety of diagnostic techniques for pulmonary lesions: systematic review, meta-analysis and network meta-analysis. Eur Respir Rev 33(173):240046. 10.1183/16000617.0046-2024 39293856 PMC11409058

[23] Lentz RJ, Frederick-Dyer K, Planz VB et al (2025) Navigational bronchoscopy or transthoracic needle biopsy for lung nodules. N Engl J Med 392(21):2100–2112. 10.1056/NEJMoa2414059 40387025 PMC12640718

[24] Paez R, Lentz RJ, Duke JD et al (2025) Robotic versus electromagnetic bronchoscopy for peripheral pulmonary lesions: a randomized trial (RELIANT). Am J Respir Crit Care Med 211(9):1644–1651. 10.1164/rccm.202409-1846OC 40460390 PMC12432399

[25] Moher D, Shamseer L, Clarke M et al (2015) Preferred reporting items for systematic review and meta-analysis protocols (PRISMA-P) 2015 statement. Syst Rev 4(1):1. 10.1186/2046-4053-4-1 25554246 PMC4320440

[26] Page MJ, McKenzie JE, Bossuyt PM et al (2021) The PRISMA 2020 statement: an updated guideline for reporting systematic reviews. BMJ 372:n71. 10.1136/bmj.n71 33782057 PMC8005924

[27] Stroup DF, Berlin JA, Morton SC et al (2000) Meta-analysis of observational studies in epidemiology (MOOSE): a proposal for reporting. JAMA 283(15):2008–2012. 10.1001/jama.283.15.2008 10789670

[28] Sterne JAC, Hernán MA, Reeves BC et al (2016) ROBINS-I: a tool for assessing risk of bias in non-randomised studies of interventions. BMJ 355:i4919. 10.1136/bmj.i4919 27733354 PMC5062054

[29] Guyatt GH, Oxman AD, Vist GE et al (2008) GRADE: an emerging consensus on rating quality of evidence and strength of recommendations. BMJ 336(7650):924–926. 10.1136/bmj.39489.470347.AD 18436948 PMC2335261

[30] Luo D, Wan X, Liu J, Tong T (2018) Optimally estimating the sample mean from the sample size, median, mid-range, and/or mid-quartile range. Stat Methods Med Res 27(6):1785–1805. 10.1177/0962280216669183 27683581

[31] Wan X, Wang W, Liu J, Tong T (2014) Estimating the sample mean and standard deviation from the sample size, median, range and/or interquartile range. BMC Med Res Methodol 14:135. 10.1186/1471-2288-14-135 25524443 PMC4383202

[32] DerSimonian R, Laird N (1986) Meta-analysis in clinical trials. Control Clin Trials 7(3):177–188. 10.1016/0197-2456(86)90046-2 3802833

[33] IntHout J, Ioannidis JPA, Borm GF (2014) The Hartung-Knapp-Sidik-Jonkman method for random effects meta-analysis is straightforward and considerably outperforms the standard DerSimonian-Laird method. BMC Med Res Methodol 14:25. 10.1186/1471-2288-14-25 24548571 PMC4015721

[34] Röver C, Knapp G, Friede T (2015) Hartung-Knapp-Sidik-Jonkman approach and its modification for random-effects meta-analysis with few studies. BMC Med Res Methodol 15:99. 10.1186/s12874-015-0091-1 26573817 PMC4647507

[35] Mantel N, Haenszel W (1959) Statistical aspects of the analysis of data from retrospective studies of disease. J Natl Cancer Inst 22(4):719–748. 10.1093/jnci/22.4.719 13655060

[36] Higgins JPT, Thomas J, Chandler J et al (eds) (2024) Cochrane Handbook for Systematic Reviews of Interventions version 6.5 (updated August 2024). Cochrane, London

[37] Yu Lee-Mateus A, Reisenauer J, Garcia-Saucedo JC et al (2023) Robotic-assisted bronchoscopy versus CT-guided transthoracic biopsy for diagnosis of pulmonary nodules. Respirology 28(1):66–73. 10.1111/resp.14368 36104312

[38] Fernandez-Bussy S, Yu Lee-Mateus A, Reisenauer J et al (2024) Shape-sensing robotic-assisted bronchoscopy versus computed tomography-guided transthoracic biopsy for the evaluation of subsolid pulmonary nodules. Respiration 103(5):280–288. 10.1159/000538132 38471496

[39] Fernandez-Bussy S, Funes-Ferrada R, Yu Lee-Mateus A et al (2025) Transforming lung cancer diagnosis: the role of robotic-assisted bronchoscopy in early detection and staging. Lung Cancer 206:108646. 10.1016/j.lungcan.2025.108646 40602203

[40] Fernandez-Bussy S, Vaca-Cartagena BF, Barrios-Ruiz A et al (2026) Comparing the effectiveness and safety of robotic-assisted bronchoscopy and computed tomography-guided transthoracic biopsy for evaluating pulmonary lung nodules: results from the DYSTRICT study. ERJ Open Res 12(3):00895–2025. 10.1183/23120541.00895-2025 42094232 PMC13139926

[41] McNierney D, Messick C, Reeve M et al (2026) Comparative performance of shape-sensing robotic-assisted bronchoscopy and CT-guided transthoracic biopsy in diagnosing peripheral pulmonary lesions. Lung Cancer 218:109487. 10.1016/j.lungcan.2026.109487 42275984

[42] Qafesha RM, Nasereddin A, Nassourah AL, Amro S, Erinat S, Ishreiteh HA, Nakhala M (2026) Robotic-assisted versus conventional CT-guided lung biopsy for pulmonary nodules: a systematic review and meta-analysis. J Robot Surg 20(1):608. 10.1007/s11701-026-03564-6 42319600

[43] Chaddha U, Agrawal A, Ghori U et al (2025) Safety and sample adequacy for comprehensive biomarker testing of bronchoscopic biopsies: an American Association of Bronchology and Interventional Pulmonology and International Association for the Study of Lung Cancer Clinical Practice Guideline. J Thorac Oncol 20(9):1237–1256 40419141 10.1016/j.jtho.2025.05.014

[44] Bott MJ, Toumbacaris N, Tan KS et al (2025) Characterizing a learning curve for robotic-assisted bronchoscopy: analysis of skills acquisition in a high-volume academic center. J Thorac Cardiovasc Surg 169(1):269–278. 10.1016/j.jtcvs.2024.06.018 38936600 PMC11655264

