# Supplementary material for: Robotic-assisted transbronchial biopsy versus computed tomography–guided transthoracic needle biopsy for peripheral pulmonary lesions: a systematic review and meta-analysis of direct comparative studies
Source: J Robot Surg. 2026 Sep 4;20(1):955. doi: 10.1007/s11701-026-03867-8 (PMC13545051; doi:10.1007/s11701-026-03867-8)
Supplement: Supplementary file 1 [file 11701_2026_3867_MOESM1_ESM.docx]

**Supplementary Table 1. Database and register search strategies and records retrieved.**

*All searches were run from database inception with no language restriction. Every strategy listed below was executed on 7 July 2026 and returned the record count shown; the Scopus and Web of Science result sets were exported and retained (736 and 75 records respectively) and are available on request. Field tags: [tiab]=title/abstract (PubMed); TITLE_ABS=title/abstract (Europe PMC); :ti,ab,kw=title/abstract/keyword; “/exp”=exploded controlled vocabulary. Truncation: * .*

| **Database / source (platform)** | **Dates searched** | **Search string** | **Records** |
| --- | --- | --- | --- |
| **MEDLINE (PubMed)** | Inception to 7 Jul 2026 | ("robotic assisted bronchoscopy"[tiab] OR "robotic-assisted bronchoscopy"[tiab] OR "robotic bronchoscopy"[tiab] OR "robot assisted bronchoscopy"[tiab] OR "shape sensing"[tiab] OR ssRAB[tiab] OR "robotic navigation"[tiab] OR "Ion Endoluminal"[tiab] OR "Monarch platform"[tiab] OR "Galaxy system"[tiab]) AND ("transthoracic needle"[tiab] OR "transthoracic biopsy"[tiab] OR "transthoracic core"[tiab] OR percutaneous[tiab] OR "CT guided biopsy"[tiab] OR "CT-guided"[tiab] OR CTTB[tiab] OR TTNB[tiab] OR TTNA[tiab] OR "needle aspiration"[tiab]) AND (lung[tiab] OR pulmonary[tiab] OR nodule*[tiab] OR lesion*[tiab] OR mass[tiab]) | **95** |
| **Europe PMC (MEDLINE + PMC + preprints)** | Inception to 7 Jul 2026 | (TITLE_ABS:"robotic bronchoscopy" OR TITLE_ABS:"robotic-assisted bronchoscopy" OR TITLE_ABS:"robot-assisted bronchoscopy" OR TITLE_ABS:"shape-sensing" OR TITLE_ABS:ssRAB) AND (TITLE_ABS:transthoracic OR TITLE_ABS:percutaneous OR TITLE_ABS:"CT-guided" OR TITLE_ABS:CTTB OR TITLE_ABS:TTNB) AND (TITLE_ABS:lung OR TITLE_ABS:pulmonary OR TITLE_ABS:nodule OR TITLE_ABS:lesion) | **69** |
| **Scopus (Elsevier)** | Inception to 7 Jul 2026 | TITLE-ABS-KEY(("robotic bronchoscopy" OR "robotic-assisted bronchoscopy" OR "shape-sensing" OR ssRAB) AND (transthoracic OR percutaneous OR "CT-guided" OR CTTB OR TTNB) AND (lung OR pulmonary OR nodule* OR lesion*)) | **736** |
| **Web of Science Core Collection** | Inception to 7 Jul 2026 | TS=(("robotic bronchoscopy" OR "robotic-assisted bronchoscopy" OR "shape-sensing" OR ssRAB) AND (transthoracic OR percutaneous OR "CT-guided" OR CTTB OR TTNB) AND (lung OR pulmonary OR nodule* OR lesion*)) | **75** |
| **ClinicalTrials.gov (register)** | Inception to 7 Jul 2026 | query.term = "robotic bronchoscopy" AND ("transthoracic" OR "CT-guided") AND lung (ClinicalTrials.gov API v2) | **3** |
| **Total records identified: 978 (975 databases + 3 registers)** | | |  |
| **Duplicate records removed (DOI/title match across all four databases)** | | | **184** |
| **Records screened** | | | **794** |
| **Records excluded at title/abstract screening** | | | **786** |
| **Reports assessed for eligibility (full text)** | | | **8** |
| **Reports excluded with reasons: 3 (2 conference abstracts; 1 non-robotic comparator)** | | |  |
| **Studies included** | | | **5** |

*CTTB, CT-guided transthoracic biopsy; RAB, robotic-assisted bronchoscopy; ssRAB, shape-sensing RAB; TTNA/TTNB, transthoracic needle aspiration/biopsy. Europe PMC overlaps MEDLINE and Scopus overlaps all sources; The three full-text exclusions were two conference abstracts (an NCCN abstract comparing RAB with CTTB, and a meeting abstract comparing shape-sensing RAB with transthoracic needle aspiration) and one full article comparing cone-beam CT–guided bronchoscopy (non-robotic) with CTTB. Records were de-duplicated by DOI and normalised title across all four databases.*
